# FLAG Regimen with or without Idarubicin in Children with Relapsed/Refractory Acute Leukemia: Experience from a Turkish Pediatric Hematology Center

**DOI:** 10.4274/tjh.2015.0411

**Published:** 2017-03-01

**Authors:** Şebnem Yılmaz Bengoa, Eda Ataseven, Deniz Kızmazoğlu, Fatma Demir Yenigürbüz, Melek Erdem, Hale Ören

**Affiliations:** 1 Dokuz Eylül University Faculty of Medicine, Department of Pediatric Hematology, İzmir, Turkey

**Keywords:** Relapsed/refractory leukemia, FLAG regimen, Chemotherapy, childhood

## Abstract

**Objective:**

The optimal therapy to achieve higher rates of survival in pediatric relapsed/refractory acute leukemia (AL) is still unknown. In developing countries, it is difficult to obtain some of the recent drugs for optimal therapy and mostly well-known drugs proven to be effective are used. We assessed the efficacy of the combination of fludarabine, high-dose cytarabine, and granulocyte colony-stimulating factor (FLAG regimen) with or without idarubicin (IDA) in children with relapsed/refractory acute lymphoblastic leukemia and acute myeloid leukemia.

**Materials and Methods:**

Between September 2007 and May 2015, 18 children with refractory/relapsed AL attending our center, treated with a FLAG regimen with or without IDA, were included. The primary end point was the remission status of the bone marrow sampled after the first/second course of chemotherapy. The second end point was the duration of survival after hematopoietic stem cell transplantation (HSCT).

**Results:**

Complete remission (CR) was achieved in 7 patients (38.8%) after the first cycle, and at the end of the second cycle the total number of patients in CR was 8 (42.1%). All patients in CR underwent HSCT. The CR rate in patients who had IDA in combination therapy was 28.6%, and it was 50% in patients treated without IDA (p=0.36). Mean survival duration in transplanted patients was 24.7±20.8 months (minimum-maximum: 2-70, median: 25 months), and it was 2.7±1.64 months (minimum-maximum: 0-5, median: 3 months) in nontransplanted patients. Five of them (27.7%) were still alive at the end of the study and in CR. The median time of follow-up for these patients was 33 months (minimum-maximum: 25-70 months).

**Conclusion:**

FLAG regimens with or without IDA produced a CR of >24 months in 27.7% of children with relapsed/refractory AL and can be recommended as therapeutic options prior to HSCT in developing countries.

## Abstract (tr)

**Amaç:**

Nüks/refrakter akut lösemili (AL) çocuklarda daha yüksek sağkalımı sağlayabilecek en uygun tedavi yaklaşımı halen bilinmemektedir. Gelişmekte olan ülkelerde bu hasta grubunda etkin olduğu iyi bilinen ve yakın zamanda geliştirilmiş bazı ilaçlara ulaşımda güçlük yaşanmaktadır. Biz relaps/refrakter akut lenfoblastik lösemili ve akut miyeloid lösemili çocuklarda idarubisin (İDA) eklenmiş veya eklenmemiş, fludarabin, yüksek doz sitarabin ve granülosit koloni stimüle edici faktör (FLAG tedavisi) kombinasyonunun etkinliğini değerlendirdik.

**Gereç ve Yöntemler:**

Çalışmaya Eylül 2007 ve Mayıs 2015 arasında merkezimizde izlenen, İDA eklenmiş veya eklenmemiş FLAG tedavisi verilen, 18 relaps/refrakter AL’li çocuk dahil edilmiştir. Birincil sonlanım noktası kemoterapi sonrası alınan kemik iliği örneğinin remisyon durumu ve ikinci sonlanım noktası ise hematopoetik kök hücre nakli (HKHN) sonrası sağkalım süresi olarak belirlenmiştir.

**Bulgular:**

Çocukların yedisinde (%38,8) ilk siklus, toplam olarak sekizinde (%42,1) ise ikinci siklus sonrasında tam remisyon (TR) elde edildi. TR’deki tüm hastalara HKHN yapıldı. Kombinasyon tedavisine IDA eklenmiş olan hastalarda TR oranı %28,6, İDA eklenmemiş olanlarda %50 idi (p=0,36). HKHN yapılmış hastalarda ortalama sağkalım süresi 24,7±20,8 ay (minimum-maksimum: 2-70, medyan: 25 ay), yapılmamış olanlarda 2,7±1,64 ay (minimum-maksimum: 0-5, medyan: 3 ay) idi. Bu hastaların beşi (%27,7) halen sağ ve TR’dedir. Yaşayan hastaların median izlem süresi 33 ay (minimum-maksimum: 25-70 ay) idi.

**Sonuç:**

IDA eklenmiş veya eklenmemiş FLAG tedavisi nüks/refrakter AL’li çocukların %27,7’sinde 24 aydan daha uzun süreli sağkalım sağlamıştır ve gelişmekte olan ülkelerde HKHN öncesi tedavi seçeneği olarak önerilebilir.

## INTRODUCTION

Despite the improved prognosis in pediatric acute leukemias (ALs), survival rates are low for patients with relapsed or refractory disease [1,2]. Treatment approaches for these patients are not uniform. Effective reinduction regimens are needed and it has been shown that hematopoietic stem cell transplantation (HSCT) can offer long survival times [2,3]. In developing countries, it is difficult to obtain some of the more recent drugs for optimal therapy, and mostly well-known drugs proven to be effective are used. Regimens with the combination of fludarabine (FL), cytarabine, idarubicin (IDA), and granulocyte colony-stimulating factor (G-CSF) have been widely used for poor-risk acute myeloid leukemia (AML), myelodysplastic syndrome (MDS), and relapsed or refractory acute lymphoblastic leukemia (ALL) in adults [4,5]. Pediatric series of AL cases with poor prognosis treated with these regimens are limited in the literature [6,7,8,9].

FL, a fluorinated purine analog, and high-dose cytarabine are effective in the treatment of ALs [10]. The combination of FL with cytarabine appears to have a synergistic effect. A positive correlation has been found between the intracellular level of the active metabolite of cytarabine, Ara-C 5’-triphosphate (Ara-CTP), and remission rates. FL triphosphate, the active metabolite of FL, inhibits ribonucleotide reductase and increases intracellular Ara-CTP. Administration of fludarabine prior to cytarabine may enhance the clinical efficacy of cytarabine [11]. IDA has also been added to the combination to increase the antileukemic effect [6,7,8,9]. G-CSF prior to FL may increase the efficacy of chemotherapy by increasing the fraction of leukemic cells in the S-phase [12].

The combination regimen of FL, high-dose cytarabine, and G-CSF (FLAG) with or without IDA has been used in relapsed/refractory acute AML and ALL patients since 2007 in our clinic. Our aim was to evaluate the rate of complete remission (CR) and duration of survival after HSCT with this regimen.

## MATERIALS AND METHODS

### Patients

Between September 2007 and May 2015, 18 children (15 boys and 3 girls) with refractory/relapsed AL attending our center were treated with a FLAG regimen with or without IDA. The median age at treatment was 12 years (minimum-maximum: 9 months to 17 years). Ten patients had a diagnosis of ALL (6 precursor B-cell and 4 T-cell ALL) and 8 had AML (2 AML-M2, 2 AML-M4, 1 AML-M5, 2 secondary AML, and 1 myeloid sarcoma). Of the 10 children with ALL, 3 cases were primary refractory, 3 first-relapsed, and 4 second-relapsed (all of them were refractory to the ALL relapse protocol), while of the 8 children with AML, 5 cases were first-relapsed and 3 primary refractory. One patient with myeloid sarcoma received the FLAG regimen after his first relapse, underwent allogeneic HSCT, relapsed 20 months after transplantation, and received the second course of the FLAG regimen.

At the time of treatment 13 patients had isolated bone marrow infiltration, 3 had isolated extramedullary disease, and 3 had combined disease. The extramedullary disease site was the central nervous system in 4 patients, testis in 1 patient, and lymph node in 1 patient.

All parents signed written informed consent forms before the start of the regimens.

### Treatment

Fludarabine at 30 mg/m^2^/day was administered intravenously over 30 min and cytarabine at 2 g/m^2^/day was administered intravenously over 3 h starting 3.5 h after completing the fludarabine infusion for 4 consecutive days (days 1-4). IDA was given at 12 mg/m^2^/day by a 1-h infusion for 3 consecutive days (days 2-4) starting 1 h before the cytarabine infusion [7]. G-CSF was given at 200 or 400 µg/m^2^/day from day 0 to the first day of absolute neutrophil count (ANC) of >1000/µL in 10 patients, while it was started 48 h after completion of treatment in 8 patients.

Nineteen courses and 30 cycles were administered to 18 patients. In 9 courses 1 cycle, in 9 courses 2 cycles, and in 1 course 3 cycles of treatment regimen were administered. Detailed information is given in Table 1.

IDA was not given to the previously heavily treated 12 patients to decrease the rate of cardiotoxicity. If CR could not be achieved after the first cycle, then IDA was added to the FLAG regimen.

All patients routinely received trimethoprim/sulfamethoxazole and antifungal prophylaxis. Patients with response to treatment underwent allogeneic HSCT if they had an eligible donor.

The toxicity of the regimen was assessed according to the Common Toxicity Criteria of the World Health Organization [13].

### Assessment of Response

Bone marrow examination was performed when ANC was >1000/µL or at day 30 after chemotherapy. CR was defined as the absence of physical signs of leukemia, no extramedullary blasts, no blasts in peripheral blood, <5% blasts in bone marrow (BM) with evidence of normal hematopoiesis, and no blasts in the cerebrospinal fluid. Partial remission (PR) was defined as marrow blasts between 5% and 25%. Aplasia was defined as blasts <5% in BM or peripheral blood, no extramedullary blasts, no evidence of hematopoietic regeneration, and no regeneration in peripheral blood count [14].

The primary end point was status of the bone marrow sampled after the first/second course of chemotherapy. The second end point was the duration of survival after HSCT.

Duration of survival was calculated from the start of the treatment regimen up to the last follow-up or mortality.

### Statistical Analysis

SPSS 15.0 (SPSS Inc., Chicago, IL, USA) was used for statistical analysis. Analytical characteristics were given as percentage, mean and SD, or median. Data were analyzed for statistically significant differences using the Mann-Whitney U test and the chi-square test. Group differences with p<0.05 were considered to be statistically significant. Duration of survival and time of follow-up were calculated with descriptive statistics. With a total of only 18 cases the use of further statistical methods was limited.

## RESULTS

### Treatment Response

Thirty cycles were administered in 18 patients. Age, sex, diagnosis, remission duration before relapses, treatment regimen and response, remission duration after treatment regimen, and duration of survival/outcome of patients are shown in Table 1. After the first cycle, CR was achieved in 7 (38.8%) patients, PR was achieved in 1 (5.3%), remission was not observed in 5 (26.3%), and aplasia was found in 3 (15.7%). Two patients could not be evaluated for response because of early death after the first FLAG treatment. After the second cycle, one nonresponder achieved CR. CR rate in patients who did not receive G-CSF starting with the chemotherapy was 50% and CR rate in patients who received G-CSF with the beginning of chemotherapy was 36.4%; the difference was not significant (p=0.563). CR rate in patients who had IDA in the combination therapy was 28.6% and CR rate was 50% in patients treated without IDA (p=0.360). There was no difference in CR rate according to the duration of remission before the treatment (p=0.770).

### Toxicity

All children had severe myelosuppression and were intensively supported with blood products. The median time of neutrophil recovery (>500/µL) was 24 days (minimum-maximum: 15-45), and that of platelet recovery (>20,000/µL) was 20 days (minimum-maximum: 15-73). Febrile neutropenia (FN) occurred after 26 (86.6%) cycles of regimens. FN was observed after 6 (85.7%) cycles with additional IDA and after 20 (87%) cycles without IDA. There was no difference in FN rate according to additional IDA (p=0.677). Most patients developed grade 3-4 mucositis. Seven children had transient mild hepatotoxicity (36.8%). There was no serious cardiotoxicity. Two patients (11.1%) had documented infections (blood cultures showed *Escherichia coli* and a yeast-like organism in 1 patient, and *Klebsiella pneumoniae* in 1 patient). Two patients, a primary refractory T-cell ALL patient and a relapsed AML patient with documented infection, died before the time of remission evaluation. Two patients (11.1%) had pulmonary invasive fungal infection.

### Duration of Survival

All patients in CR and one patient with AML secondary to MDS who had aplasia after the regimen, in total 9 (50%) patients, underwent subsequent allogeneic HSCT. Four patients were transplanted from matched sibling donors, 2 from matched unrelated donors, and 3 from haploidentical donors. Four patients died after HSCT; in 3 patients, the cause of death was infection. The fourth patient (case 18) relapsed after HSCT and had a second course with 3 cycles of FLAG; he was in remission after the first 2 cycles but relapsed after the third cycle and died.

Mean duration of survival in transplanted patients was 24.7±20.8 months (minimum-maximum: 2-70, median: 25 months) and it was 2.7±1.64 months (minimum-maximum: 0-5, median: 3 months) in the nontransplanted patients.

As a result, 5 (27.7%) patients who underwent HSCT are still alive and in CR. Two patients underwent allogeneic HSCT from their siblings, 2 underwent allogeneic HSCT from unrelated matched donors, and 1 underwent haploidentical HSCT from his mother. The median time of follow-up for these patients was 33 months (minimum-maximum: 25-70 months). Three were AML (one case secondary to MDS) and 2 were ALL patients.

## DISCUSSION

The treatment of children with relapsed or refractory AL is still challenging. Regimens containing FL and high-dose cytarabine with or without IDA have been used in this patient group, and the first results were published in 1996 [6]. In our study, the CR rate after 2 cycles was 42.1% (most of these patients were in CR after the first cycle), all of these patients could proceed to HSCT, and 27.7% survived. Fleischhack et al. reported a CR rate of 73.9% in patients with poor-prognosis AML; 47.8% underwent HSCT and 39.1% remained in CR [7]. In the study conducted by McCarthy et al., in a group of ALL, AML, and biphenotypic AL patients using the FLAG regimen the CR rate was 70%; 68.4% of the patients underwent HSCT and 36.8% were alive at the end of the study [15]. Tavil et al. from Turkey presented the results of 25 relapsed/refractory AL patients. The CR rate was 60%, 49% of their patients could proceed to HSCT, and 20% survived [8]. Yalman et al., also from Turkey, reported a CR rate of only 17.6% in 17 poor-prognosis AL patients; 2 underwent HSCT and only 1 child with a previous HSCT survived after donor lymphocyte infusion [9].

The CR rate in our patients who had IDA in combination therapy was 28.6% and it was 50% in patients treated without IDA; the difference was not statistically significant. Patients who received IDA-FLAG were mostly those who had refractory disease. This might be the reason for the lower response rate.

All of our patients experienced severe myelosuppression, FN developed after 86.6% of the cycles, and 2 patients (11.1%) died early with infection, shortly after chemotherapy (15^th^ and 17^th^ days). The addition of IDA to the FLAG regimen did not change the risk of FN. Invasive fungal infection was observed in a total of 3 patients (16.6%). The reported toxicity of these regimens is similar to rates reported in the literature [8,15].

In some recent studies, it was demonstrated that with the addition of agents like liposomal forms of daunorubicin and doxorubicin instead of IDA to treatment regimens containing FL and high-dose cytarabine, CR can be achieved in higher rates with less systemic toxicity in children with refractory/relapsed AL [16,17]. In developing countries such as Turkey, liposomal forms of these anthracyclines are not available and cannot be used due to economic reasons.

Because of the increased use of unrelated and haploidentical donors nowadays, even when a suitable family donor is lacking, the chance of transplantation with alternative stem cell sources in a short time after CR is better. Therefore, achieving CR in poor-prognosis AL with effective treatment regimens may result in better outcomes. Five of our patients achieved a CR of >24 months after HSCT.

The role of G-CSF in the management of relapsed/refractory AL has been tested widely and remains controversial [18]. Most of the trials demonstrated a modest reduction in the duration, but not the depth, of neutropenia [16,18,19]. The effects of G-CSF on duration of survival, incidence of severe infection, and duration of hospitalization are variable, but in developing countries, the death rates due to FN are higher than in developed countries, and G-CSF given with chemotherapy or after chemotherapy is still common. Even though a trend towards an increased incidence of relapses with G-CSF treatment in children with AML that overexpress the differentiation-defective G-CSFR isoform IV has been reported, the number of these cases is very low and G-CSF continues to be a part of the FLAG regimen [16,20]. We used G-CSF in all of our patients since the FN risk is high in our clinic. We did not find any statistically significant difference in CR rate whether we started G-CSF at day 0 or after completion of chemotherapy.

## CONCLUSION

In conclusion, FLAG regimens with or without IDA produced a CR of >24 months in 27.7% of children with refractory/relapsed AL and can be recommended as a therapeutic option prior to HSCT with appropriate supportive measurements in developing countries.

## Figures and Tables

**Table 1:**
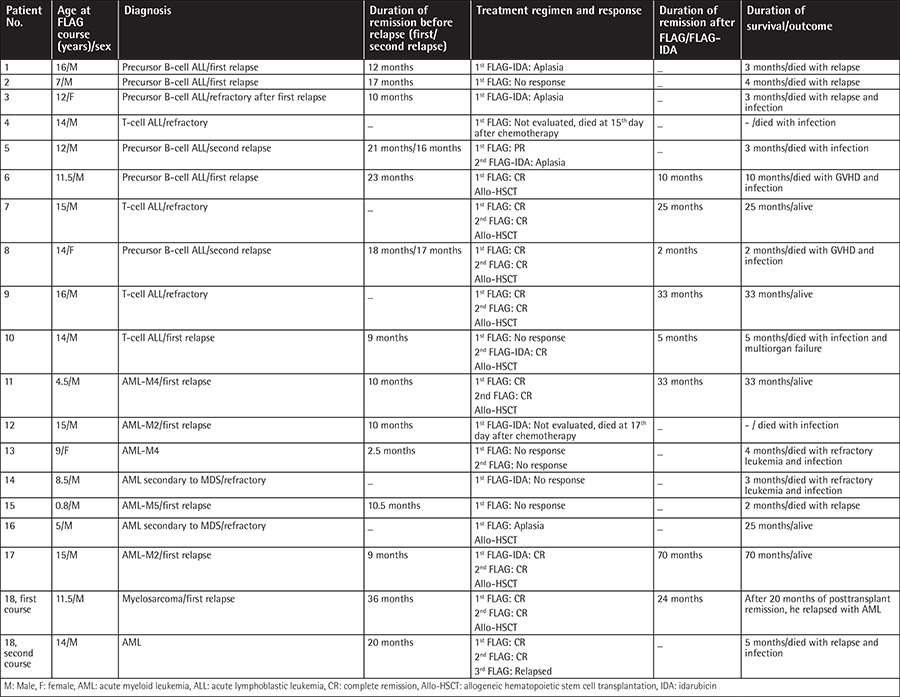
Patient characteristics, treatment regimen, response to treatment, duration of survival, and outcome.
